# Dual regulation of the cGAS-STING pathway: new targets and challenges for subtype-specific immunotherapy in breast cancer

**DOI:** 10.3389/fonc.2025.1619097

**Published:** 2025-06-10

**Authors:** Hanchu Xiong, Rongxue Li, Lixian Yang, Yucheng Li, Xiao Ma

**Affiliations:** Cancer Center, Department of Radiation Oncology, Zhejiang Provincial People’s Hospital, Affiliated People’s Hospital, Hangzhou Medical College, Hangzhou, Zhejiang, China

**Keywords:** cGAS, STING, breast cancer, tumor microenvironment, innate immunity

## Abstract

Research on immune regulation mechanisms in breast cancer is crucial for breaking through therapeutic bottlenecks. This paper comprehensively reviews the dual roles of the cGAS-STING pathway in Luminal, HER2+, and triple-negative breast cancer (TNBC): its activation can enhance antitumor immunity, but chronic activation may lead to immunosuppression. By integrating molecular mechanisms, clinical translation, and subtype-specific strategies, it provides new directions for precision immunotherapy.

## 1 Introduction

Breast cancer, the most frequently occurring malignant tumor among women globally, poses a serious threat to patients’ health and quality of life and is also one of the leading causes of cancer-related deaths (1, 2). Its complexity lies in its heterogeneity, being composed of different subtypes with varying biological behaviors, morphological characteristics, and treatment responses. Based on molecular features, breast cancer is mainly divided into four subtypes: Luminal type (60-70%): including Luminal A (HR+/HER2-/low Ki-67) and Luminal B (HR+/high Ki-67); HER2+ type (20%): driven by HER2 overexpression leading to aggressive growth; Triple-negative breast cancer (TNBC) (10-15%): ER/PR/HER2-negative, with a high risk of recurrence and metastasis (3, 4). According to the NCCN treatment guidelines, endocrine therapy is a common treatment for ER-positive breast cancer patients, and anti-HER2 targeted therapy is recommended for HER2-positive patients (5). However, there is currently no specific targeted therapy for TNBC. Despite significant progress in diagnosis and treatment, including multi-modal strategies such as surgery, chemotherapy, radiotherapy, endocrine therapy, and targeted therapy, 20%-30% of patients may still develop metastatic disease (6, 7). Recently, immune checkpoint inhibitors (e.g., anti-PD-1/PD-L1) have achieved breakthroughs in TNBC, with the KEYNOTE-355 trial showing that pembrolizumab combined with chemotherapy extended progression-free survival (PFS) from 5.6 to 9.7 months in advanced TNBC patients (6, 8, 9). However, immune therapy still faces significant challenges. The tumor microenvironment varies greatly among subtypes, with high T-cell infiltration but strong immunosuppression in TNBC; HER2+ type has STING pathway inhibition leading to trastuzumab resistance, and the Luminal type has AKT1 overactivation weakening immune response; systemic immune activation may also trigger cytokine storms or autoimmune reactions (10–12). However, due to tumor heterogeneity and the development of drug resistance, the long-term effects of these treatments are often limited. Therefore, identifying new therapeutic targets and developing effective strategies for metastatic breast cancer is crucial for improving patient prognosis and survival rates.

Immunotherapy has made significant strides in cancer treatment. Immune checkpoint inhibitors targeting PD-1/PD-L1 show promise across multiple malignancies (13, 14). As tumor immunotherapy gains precision and efficacy, interest in developing new approaches is rising. The cancer-immune system interaction is intricate and dynamic. While the immune system can combat tumors by identifying and eliminating abnormal cells, tumors can evade this response through mechanisms like PD-L1 upregulation and Treg recruitment (15, 16). cGAS is a cytosolic innate immune sensor of double-stranded DNA (dsDNA), which interacts with the sugar-phosphate backbone of dsDNA via positively charged amino acid residues, a process further facilitated by a conserved zinc ribbon (17). Activated cGAS synthesizes cGAMP using ATP and GTP, inducing conformational changes in the endoplasmic reticulum-resident STING protein and promoting its trafficking from the endoplasmic reticulum to the Golgi apparatus (18). The most common mechanism by which STING enhances antitumor immune function is through the induction of interferon and inflammatory cytokine production, thereby activating cytotoxic CD8+ T cells to promote adaptive immune responses (19). The cGAS-STING pathway, linking DNA damage to immune responses, plays a dual role in breast cancer (20). It can both enhance antitumor immunity and, when chronically activated, potentially drive immunosuppression (21). STING agonists can enhance antitumor immune responses, their combination with radiotherapy can boost complete response rates in TNBC (22). In HER2+ breast cancer, STING activation reverses trastuzumab resistance, and combination therapy with DS-8201 can extend median progression-free survival (mPFS). In the Luminal subtype, STING expression correlates with macrophage infiltration, yet chronic activation may expand Tregs, blunting therapeutic effects (23, 24).

**Figure 1:**
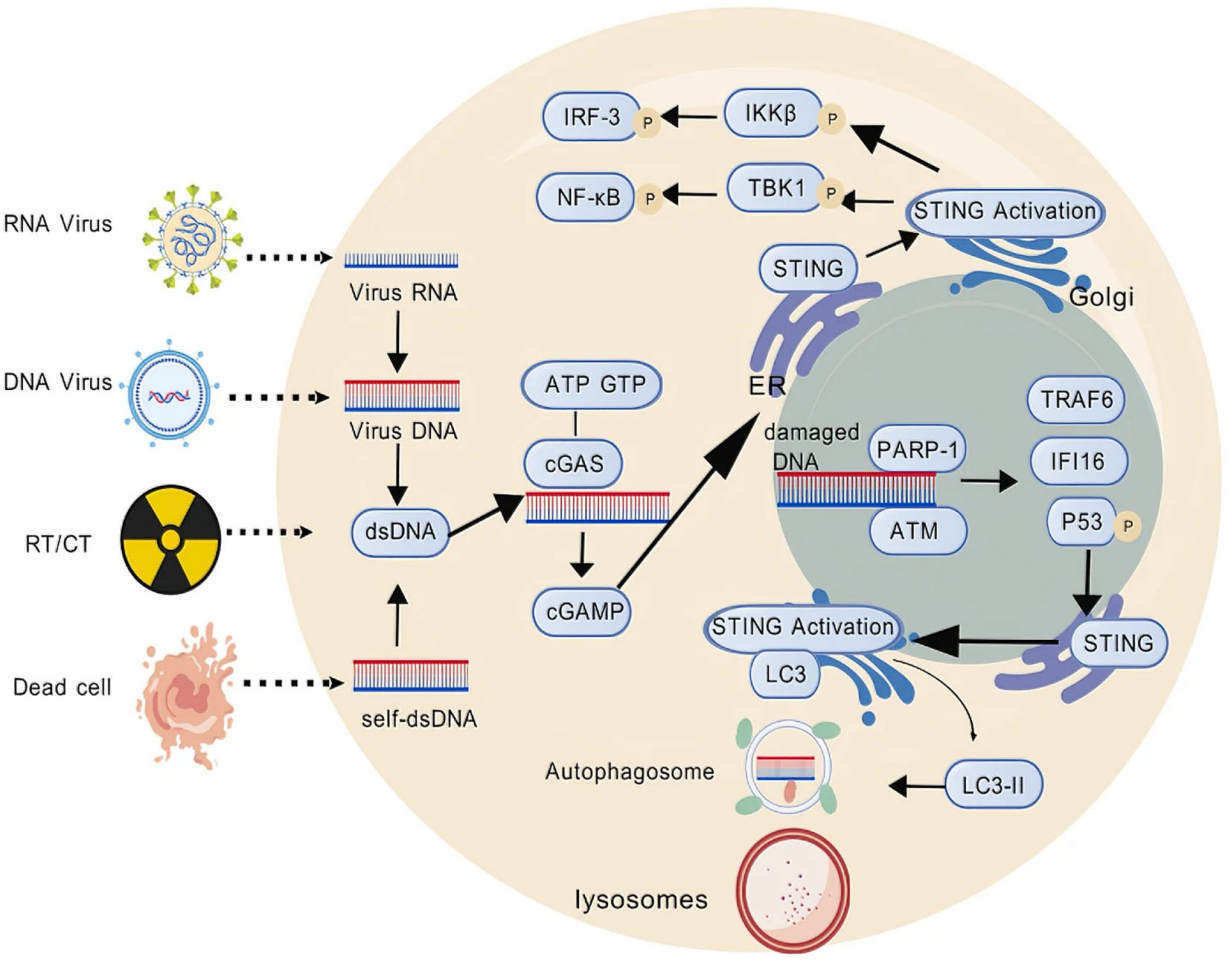
Schematic of the cGAS–STING pathway. cGAS-dependent STING pathway: dsDNA activates cGAS, which catalyzes the synthesis of cGAMP, subsequently leading to IRF3 and NF-κB-driven expression of type I interferons and pro-inflammatory factors. cGAS-independent STING pathway: damaged DNA-induced PARP-1 and ATM are recruited and promote the assembly of a STING signaling complex comprising P-p53, IFI16 and TRAF6, which acts as a potential autophagy receptor to promote the lipidation of microtubule-associated LC3, inducing autophagy to eliminate invading pathogens and cytosolic DNA. This figure was generated by GDP (29).

**Figure 2:**
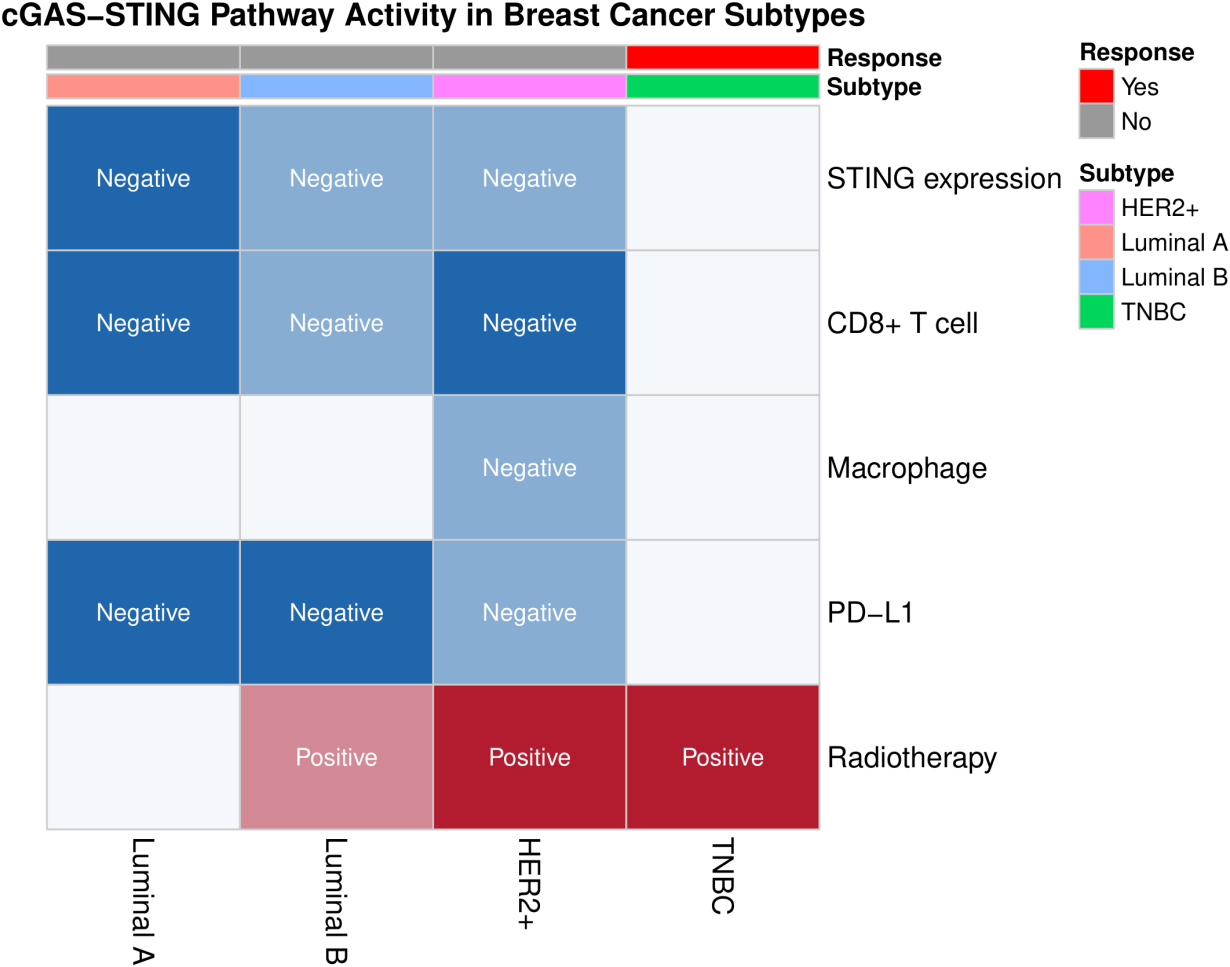
Heatmap of key immunological biomarkers (e.g., STING expression, CD8+ T cell infiltration, macrophage infiltration, PD-L1 expression) and immunotherapy responses in different subtypes of breast cancer. TNBC shows higher radiotherapy sensitivity (marked in red) and unique potential for immunotherapy, contrasting with the suppressed STING pathway activity (marked in blue) in Luminal subtypes. This figure was generated by the R package “ggplot2” (32).

cGAS-STING activation begins when cGAS detects cytosolic dsDNA, such as damage from radiotherapy or chemotherapy. This triggers a cascade: cGAS produces cGAMP, which activates STING, leading to IRF3 and NF-κB-driven expression of type I interferons and pro-inflammatory factors (25, 26). Beyond the classical cGAS-STING pathway characterized by IFN-I and other cytokine expression, non-canonical STING activation pathways should also be noted. STING can directly activate autophagy independently of TBK1-IRF3 and classical autophagy signaling molecules (27). When activated STING translocates to the endoplasmic reticulum-Golgi intermediate compartment (ERGIC), it acts as a potential autophagy receptor, using ERGIC as the primary membrane source to promote the lipidation of microtubule-associated LC3, inducing autophagy to eliminate invading pathogens and cytosolic DNA (28) (**Figure 1**). Several STING agonists are now in clinical trials, showing potential in breast cancer. For example, in a phase I trial (NCT03937141) targeting advanced/metastatic solid tumors or lymphomas, researchers explored the combined therapeutic effects of the STING agonist ADU-S100 (MIW815) and the PD-1 inhibitor pembrolizumab. Results showed that this combination therapy could significantly enhance antitumor immune responses in certain patients, as evidenced by an increase in tumor-infiltrating lymphocytes (TILs) (30). MK-1454 in a phase II trial for HER2+ breast cancer extended mPFS by 3.1 months when combined with trastuzumab and chemotherapy, though with a 18% incidence of grade 3+ febrile neutropenia (31). These findings indicate that combining STING agonists with existing therapies may overcome resistance but require further safety optimization. By finely tuning cGAS-STING activity, we can enhance breast cancer immunogenicity and the efficacy of immune checkpoint inhibitors, offering more effective strategies for patients. Moreover, the link between cGAS-STING activation and genetic instability in breast cancer opens new avenues for personalized treatment strategies tailored to specific genetic backgrounds (**Figure 2**).

This review systematically summarizes the pivotal roles of the cGAS-STING signaling pathway in breast cancer initiation, progression, drug resistance, and therapeutic responses, as well as its profound impact on tumor advancement and microenvironmental remodeling. The article delves into the intricate interplay between the activation status of the cGAS-STING pathway and the tumor immune microenvironment across distinct breast cancer subtypes, elucidating how these interactions may lead to divergent clinical outcomes—either beneficial or detrimental. Furthermore, the review critically examines current research directions and challenges associated with the cGAS-STING axis in breast cancer, while emphasizing recent breakthroughs in cGAS-STING-related studies. By integrating these insights, this work aims to deepen the mechanistic understanding of researchers and clinicians regarding the dual roles of cGAS-STING in breast cancer pathogenesis, ultimately paving the way for novel precision therapeutic strategies tailored to the molecular heterogeneity of the disease.

## 2 The cGAS-STING pathway and breast cancer

Breast cancer encompasses major subtypes, including Luminal, HER2+, and TNBC, each of which exhibits significant differences in response to treatment. The cGAS-STING pathway activates antitumor immunity by detecting cytoplasmic DNA, but its effects vary among subtypes. In the pathogenesis of breast cancer, the activation of the cGAS-STING signaling pathway plays a crucial role in enhancing antitumor immune responses, thereby suppressing the progression and metastasis of breast cancer, offering new potential strategies for immunotherapy in breast cancer (33). Cancer immunotherapy (ICI) is a key and rapidly evolving treatment modality that stands alongside surgical intervention, cytotoxic chemotherapy, radiotherapy, and targeted therapy (34). Thus, it represents the fifth pillar of cancer management. As our understanding of the molecular mechanisms and roles of the cGAS-STING pathway in breast cancer deepens, the development of new immunotherapeutic approaches is likely to benefit.

In recent years, research on the cGAS-STING pathway in breast cancer has made significant progress, and its regulatory mechanisms and clinical significance have become increasingly clear. Chen et al. systematically analyzed the prognostic value of cGAS-STING related genes (CSRG) in breast cancer patients by obtaining 1,087 breast cancer samples and 179 normal breast tissue samples from The Cancer Genome Atlas (TCGA) and Genotype Tissue Expression (GTEX) databases, identifying 35 CSRGs. Further refinement using Cox regression identified 11 differentially expressed genes (DEGs) associated with prognosis, constructing a machine learning-based risk assessment and prognostic model. The study results indicate that this risk model can effectively predict the survival and prognosis of breast cancer patients, with patients having a low-risk score showing significantly better overall survival (OS) compared to those with a high-risk score (35).

Additionally, the study found a significant correlation between the risk score and tumor-infiltrating immune cells, immune checkpoints, and responses to immunotherapy, providing a new perspective for precision treatment and prognostic evaluation of breast cancer. The expression of the STING protein is closely related to the tumor microenvironment in breast cancer. In breast cancer tissues, the expression levels of STING are positively correlated with the infiltration of tumor-associated macrophages (TAMs), indicating that STING may affect tumor progression by regulating the infiltration of TAMs (36). Ka et al. showed that in breast cancer, NR1D1 promotes the accumulation of dsDNA fragments induced by DNA damage, activating the cGAS-STING signaling pathway, thereby increasing the production of type I IFN and downstream chemokines CCL5 and CXCL10 (37). Totis et al. have indicated that radiotherapy can trigger the activation of the cGAS-STING axis through the induction of cytoplasmic dsDNA fragments (38). The activation of cGAS-STING initiates type I interferon-mediated innate immune signaling, which is instrumental in eradicating malignant tumors (18). These findings reveal the potential of cGAS-STING pathway activation for anti-tumor immunity. Recently, there has been a growing interest in the activation of the cGAS-STING pathway through nanomaterials, providing new strategies for immunotherapy in breast cancer. However, Qin and others have shown that even in the presence of cytoplasmic DNA accumulation, hypoxia can induce an immunosuppressive phenotype in tumor cells (39). The mechanisms by which tumor cells suppress the activation of the cGAS-STING pathway induced by hypoxia to evade immunity are largely unclear. Research has shown that hypoxia stimulates JNK1/2-mediated phosphorylation of phosphoenolpyruvate carboxykinase 1 (PCK1) at S151, a phosphorylation that triggers the interaction between PCK1 and cGAS. PCK1 associated with cGAS competitively consumes GTP, a substrate shared by PCK1 and cGAS. Consequently, PCK1 inhibits GTP-dependent cGAS activation and subsequent STING-promoted immune cell infiltration and activation in the tumor microenvironment, thereby promoting tumor growth in mice. Blocking PCK1 function in combination with anti-PD-1 antibody therapy exhibits an additive therapeutic effect on tumor growth (39). Furthermore, PCK1 S151 phosphorylation is negatively correlated with cGAS-STING activation and patient survival rates in human breast cancer specimens.

These findings reveal the complex mechanisms of the cGAS-STING pathway in breast cancer, showing both anti-tumor potential and the possibility of promoting tumor progression and drug resistance under certain conditions. Further exploration is needed to clarify the specific roles of the cGAS-STING pathway in different breast cancer subtypes and to develop targeted therapeutic strategies. In particular, modulating the cGAS-STING pathway to overcome immune suppression and drug resistance will be an important direction for breast cancer immunotherapy.

### 2.1 The role of the cGAS-STING pathway in luminal breast cancer

Luminal-type breast cancer, defined by molecular traits and hormone receptor expression, is linked to a better prognosis (4). It’s divided into Luminal A and B subtypes based on ER, PR levels and HER2 status. Characterized by high ER and PR expression and low or negative HER2 expression, these cancers often respond well to hormone therapy due to the presence of ER and PR. They typically have lower proliferation rates and slower progression compared to HER2-positive or TNBC subtypes (40, 41). However, Luminal-type patients still face recurrence and metastasis risks, especially early after treatment, and may develop endocrine resistance, limiting therapeutic efficacy (42). The cGAS-STING pathway’s role and regulatory mechanisms in Luminal breast cancer are a hot research topic. Recent studies indicate it significantly impacts tumor immunoregulation and drug resistance, with a dual role in the Luminal subtype (43, 44). Thus, in addition to endocrine therapy, combining other treatments like chemotherapy, radiotherapy, targeted therapy, and immunotherapy may be needed to enhance outcomes and survival rates in Luminal-type breast cancer patients.

In Luminal breast cancer, the activation status of the cGAS-STING pathway has a complex interplay with the tumor immune microenvironment (45). As analyzed by Liu et al., analyzing STING and CD68 expression in breast cancer tissues reveals links between their expression and immune cell infiltration. Results show a significant correlation between positive STING expression, HER2 positivity, and the Luminal subtype. Notably, in Luminal A and B breast cancer patients, STING expression correlates positively with macrophage infiltration (46). Macrophages, key immune cells in the tumor microenvironment, are closely related to tumor immune responses and prognosis. These findings offer new insights into understanding the immune microenvironment and treatment responses in breast cancer. In ER+HER2- breast cancer, the abnormal inactivation of the cGAS-STING pathway is linked to endocrine therapy resistance. Zhang’s team found that in drug-resistant cells, over-activation of AKT1 kinase hinders the formation of the TBK1/STING/IRF3 complex, reducing cytoplasmic DNA sensing and suppressing cGAS-STING activation (47). Notably, cGAS-STING inhibition and AKT1 activation form a positive feedback loop, worsening drug resistance. Animal experiments show that combining AKT1 inhibitors with STING agonists can disrupt this cycle. This approach activates innate (e.g., dendritic cells) and adaptive immunity (e.g., cytotoxic T cells), significantly curbing tumor growth and offering a new strategy to reverse drug resistance.

While cGAS-STING activation may enhance antitumor immunity, sustained activation can pose tumorigenic risks. For example, STING signaling can promote the expansion of regulatory T cells (Tregs) by inducing the secretion of immunosuppressive factors like IL-6, ultimately leading to immune evasion (48, 49). This dual-edged effect suggests the need for precise pathway regulation. On one hand, restoring its normal function in drug-resistant patients is required to enhance immune surveillance. On the other hand, over-activation induced immunosuppressive microenvironments must be avoided (50, 51). It’s crucial to deeply understand the spatiotemporal specific regulatory mechanisms of the cGAS-STING pathway in the Luminal subtype. Research priorities should include: dynamic changes in pathway activity in different molecular subtypes (Luminal A/B), how tumor-stromal cell crosstalk affects pathway function, developing combination therapies based on pathway status such as sequential STING agonist and immune checkpoint inhibitor or AKT inhibitor use.

In summary, precise cGAS-STING pathway activity regulation is key for balancing immune activation and suppression. Future research should further explore its specific mechanisms in Luminal breast cancer and drug-based optimization of its antitumor effects.

### 2.2 The role of the cGAS-STING pathway in HER2+ breast cancer

HER2+ breast cancer is characterized by the overexpression of HER2, and targeted therapies (e.g., trastuzumab, DS-8201) have significantly improved patient prognoses (52, 53). However, drug resistance and an immunosuppressive tumor microenvironment remain clinical challenges. Recent studies indicate that abnormal regulation of the cGAS-STING pathway interacts with HER2 signaling, jointly influencing treatment responses and immune evasion (54).

Recent studies have shown that HER2-targeted therapy resistance is associated with pathway inhibition. Cai et al. found that the tumor microenvironment in HER2+ breast cancer patients with resistance shows significant immunosuppression, which is closely related to reduced activity of the cGAS-STING pathway (55). The immune-related prognostic index (IRPI) they constructed shows that patients with high IRPI scores have poorer prognoses, further confirming that cGAS-STING pathway inhibition is a key mechanism driving immune evasion in trastuzumab-resistant breast cancer. Notably, combining STING agonists with DS-8201 can reverse the immunosuppressive phenotype and significantly inhibit the progression of resistant tumors in preclinical models (56). Wu et al. found that HER2 overexpression can weaken antitumor immune responses by disrupting cGAS-STING signaling in Colorectal cancer. Anti-HER2 therapies (e.g., trastuzumab) may partially restore cGAS-STING pathway function, activating the tumor-killing activity of CD8+ T cells and NK cells. This suggests a synergistic effect between HER2-targeted therapy and immune modulation (57).

### 2.3 The role of the cGAS-STING pathway in TNBC

TNBC is a unique breast cancer type, lacking ER, PR, and HER2 expression. It accounts for 10-15% of breast cancer cases and is highly aggressive, metastatic, and associated with a poor prognosis (58, 59). Due to the lack of effective targeted therapies, TNBC patients face a higher risk of recurrence (60). Recent studies indicate that the cGAS-STING pathway plays a dual role in TNBC by modulating immune responses and the tumor microenvironment (61). Nanotechnology is emerging as a new approach for precise regulation of this pathway (62, 63).

In TNBC, immunological activation is linked to therapeutic enhancement. When cytosolic DNA (e.g., from radiotherapy/chemotherapy-induced damage) activates the cGAS-STING pathway, it triggers type I interferon signaling via the STING-TBK1-IRF3 axis, recruiting CD8+ T cells and NK cells to enhance antitumor immunity (64, 65). Preclinical studies show that TNBC patients with high STING expression have a prolonged progression-free survival by 6 months compared to those with low expression, indicating its potential as a prognostic biomarker. For example, Liu et al. found that radiotherapy - induced DNA damage significantly inhibits tumor growth in TNBC mouse models by activating the cGAS-STING pathway (66). Xu et al. pointed out that activating the cGAS-STING pathway can enhance the immunogenicity of TNBC cells and improve the efficacy of immune checkpoint inhibitors (67). They developed a ruthenium(II) arene complex C5 based on ferritin and constructed a C5-AFt nanoparticle delivery system, which inhibits the growth and metastasis of TNBC by inducing ferroptosis via mitochondrial damage and activating the cGAS-STING pathway. On the other hand, the cGAS-STING pathway can promote tumor immune evasion under microenvironmental stress. For instance, in triple-negative breast cancer cells, when there is an error in chromosome segregation, the cGAS-STING signaling pathway is activated. The activation of this pathway further triggers the non-classical NF-κB signaling pathway, leading to the release of IL-6 and activating the IL-6/STAT3 signaling pathway, ultimately promoting the survival and development of drug resistance of triple-negative breast cancer cells (68).

In recent years, nanomaterials have become a research hotspot in the treatment of TNBC due to their precise delivery and multifunctional characteristics. Many studies have been committed to the activation of the cGAS-STING pathway based on nanotechnology. The core challenge in TNBC treatment lies in overcoming the immunosuppressive microenvironment and enhancing drug penetration, and the precise delivery and multifunctional characteristics of nanomaterials provide a breakthrough direction for this. The oxygen self-supplying nanoradiosensitizer ALFM developed by Wang et al. enhances the antitumor immune response and improves radioimmunotherapy for TNBC by activating immunogenic cell death (ICD) and the cGAS-STING signaling pathway (69). The study by Kim et al. has shown that cannabidiol (CBD) can stimulate PD-L1 expression in TNBC cells and significantly activate the cGAS-STING pathway (70). The combination of CBD and anti-PD-L1 antibodies enhances the antitumor immune response. These two examples both illustrate that the activation of the cGAS-STING pathway enhances immunogenicity. Moreover, the activation of the cGAS-STING pathway is also associated with the remodeling of the tumor microenvironment and the enhancement of drug penetration. For example, the study by Zhou et al. developed a zinc-copper bimetallic nanoplatform coated with polydopamine (CZP NPs), which can effectively induce photothermal-enhanced copper deposition and activate the cGAS-STING signaling pathway, thereby reversing the immunosuppressive tumor microenvironment in TNBC (71). Photothermal therapy significantly enhances these effects, and the combination of CZP NPs with αPD-L1 markedly boosts antitumor immunity and suppresses tumor growth. Some researchers have combined losartan, a STING agonist, and a PD-L1 inhibitor to form an intelligent nanosystem. Losartan is released in the tumor microenvironment, where it degrades the extracellular matrix to enhance the penetration of immunotherapeutic drugs (72). Subsequently, the reactive oxygen species generated by photosensitizers ensure the targeted release of drugs, activate the cGAS-STING pathway, and enhance the immune response.

With the rapid development of nanotechnology and biomaterials science, new materials are constantly emerging, bringing more opportunities for the treatment of TNBC. In the field of TNBC, many materials have demonstrated antitumor efficacy by activating the cGAS-STING pathway. Specifically, these materials can influence the growth of TNBC cells. For instance, under growth-restricted conditions, activation of the cGAS-STING pathway helps breast cancer cells survive, while inhibition of this pathway triggers autophagy-dependent cell death mechanisms. Additionally, these materials can modulate the tumor microenvironment. For example, by promoting photothermal-enhanced copper deposition and activating the cGAS-STING signaling pathway, they can alter the immunosuppressive state of TNBC, enhance antitumor immune responses, and inhibit tumor growth. Moreover, they can activate immune responses, such as enhancing the immunogenicity of TNBC cells and improving the therapeutic efficacy of immune checkpoint inhibitors by activating the cGAS-STING pathway. This ultimately brings more effective treatment options for TNBC patients. Looking to the future, the in-depth integration of materials science with immunotherapy and other multidisciplinary fields is expected to provide more efficient and precise treatment options for TNBC patients. On one hand, new materials can achieve precise drug delivery, increasing the concentration of drugs in tumor tissues and reducing toxicity to normal tissues. On the other hand, through rational design and functional modification, materials can possess multiple functions, such as simultaneously activating the cGAS-STING pathway, modulating the tumor microenvironment, and enhancing immune responses, to maximize the synergistic antitumor effect.

### 2.4 Involvement of cGAS-STING pathway in stemness, metastasis and drug resistance of breast cancer

In Luminal breast cancer, the inactivation of the cGAS-STING pathway and the overactivity of AKT kinase form a vicious cycle, which is a significant cause of endocrine therapy resistance and metastasis in patients (47). Team Dengrong from Sun Yat-sen University has found that the cGAS-STING pathway activity is significantly reduced in Luminal breast cancer cells, while AKT kinase is abnormally active. AKT directly interferes with the key molecule TBK1 downstream of STING, causing the STING-IRF3 signaling pathway to “malfunction,” thereby inhibiting the immune system’s ability to produce type I interferons and allowing tumor cells to evade immune attacks, ultimately rendering drugs (such as tamoxifen) gradually ineffective (47). For this mechanism, Team Dengrong confirmed that combination therapy strategies have shown significant potential: STING agonists (such as ADU-S100) can reactivate immune surveillance functions, while AKT inhibitors (such as Capivasertib) can block the abnormal signaling of the enzyme, and their synergistic effect not only reverses drug resistance but also transforms immune “cold tumors” into “hot tumors,” enhancing T cell infiltration. Meanwhile, another Team Ang Zheng found that SNORA47 affected stemness and chemotherapy sensitivity of Luminal breast cancer cells via EBF3/RPL11/c-Myc axis, providing a new direction for precision therapy of Luminal breast cancer patients (73).

Studies have shown that resistance to HER2-targeted therapies in HER2+ BC is often associated with the abnormal activation of mesenchymal HER2+ cancer stem cells, Team Serenella M Pupa found that a consistent enrichment of CD36 in HER2+ breast cancer stem cells from all tested resistant cell models that mechanistically occurs via Wnt signaling pathway activation (74). Consistently, dual blockade of CD36 and HER2 increased the efficacy of anti-HER2 drugs favoring the transition of stem cells into therapy-sensitive epithelial state. In addition, other preclinical experiments have shown that the combination of STING agonists (such as ADU-S100) with HER2-targeted drugs (such as trastuzumab or DS-8201) can significantly reduce the proportion of cancer stem cells and inhibit the clonogenic ability of drug-resistant cells, suggesting that STING activation may reverse resistance by remodeling the TME and regulating stem cell properties (75).

In TNBC, the abnormal regulation of the cGAS-STING pathway is closely related to tumor immune evasion and therapeutic drug resistance. Researchers have found that TNBC cells activate the phosphorylation of the metabolic enzyme ADSL under hypoxic conditions, promoting the abnormal accumulation of its metabolic product, fumarate (48). Fumarate can directly bind to and inhibit the activity of the STING protein, blocking the cGAMP-mediated STING-IRF3 signal transduction, leading to a decrease in the secretion of type I interferons, thereby weakening the anti-tumor immune response and causing drug resistance to immune therapies (48). Since Mn^2+^ has great potential for activating the cGAS‐STING signaling pathway to generate antitumor immune responses, Team Haisheng reported that microneedles loaded with sparfloxacin and Zinc-Manganese sulfide nanoparticles could significantly suppress tumor growth, thereby significantly enhancing the tumor infiltration and cytotoxic effects of CD4+/CD8+ T cells and strongly inhibiting the lung metastasis of TNBC cells (76).

## 3 Conclusion

**Figure 3:**
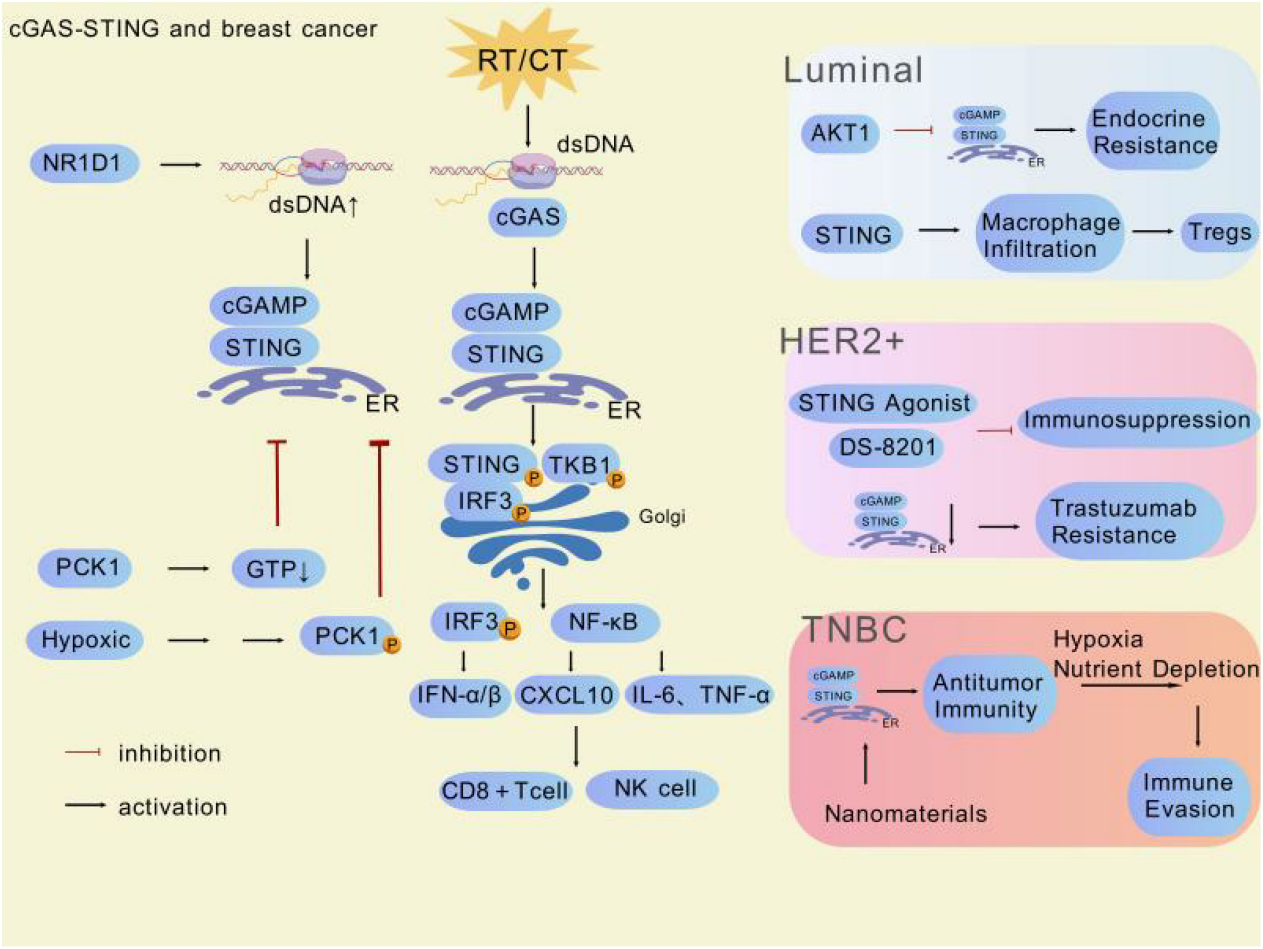
Multiple factors and potentially clinically valuable drugs are involved in regulating the cGAS-STING pathway in different subtypes of breast cancer. This figure was generated by GDP (29).

After reviewing and organizing a large number of relevant studies, we have gained a comprehensive understanding of the role of the cGAS-STING pathway in different breast cancer subtypes (**Table 1**). Overall, this pathway plays a crucial role throughout the course of breast cancer, with its activation state closely linked to the tumor immune microenvironment. On one hand, activation of the pathway can enhance antitumor immune responses, opening new avenues for breast cancer immunotherapy. On the other hand, its sustained activation in certain contexts may lead to immunosuppression and drug resistance, negatively impacting treatment outcomes (**Figure 3**). Specifically, in Luminal breast cancer hyperactivation of AKT kinase inhibits the formation of the STING-IRF3 complex by binding to TBK1, blocking IFN-I secretion and impairing immune surveillance. Concurrently, STING signaling inactivation further promotes AKT phosphorylation, forming a vicious cycle that drives endocrine therapy resistance. Endocrine resistance in Luminal breast cancer (particularly the ER+/HER2- subtype) remains a major research focus. Deng Rong’s team validated through clinical samples and humanized mouse models that combining STING agonists (e.g., ADU-S100) with AKT inhibitors (e.g., Capivasertib) breaks this cycle, significantly suppressing resistant tumor growth and promoting CD8+ T-cell infiltration, thereby converting “cold tumors” into “hot tumors”. However, chronic STING activation may upregulate PD-L1 expression via the IRF3 signaling axis and induce regulatory T-cell expansion, leading to an immunosuppressive microenvironment. The resistance mechanisms of STING agonists in Luminal breast cancer are complex, involving signaling pathway imbalances, metabolic abnormalities, and immune microenvironment remodeling, necessitating further research. In HER2-positive breast cancer, STING agonists (e.g., ADU-S100) activate the cGAS-STING pathway to promote IFN-I secretion, enhancing APC maturation and cross-presentation. However, PI3K/AKT pathway hyperactivity driven by PIK3CA mutations inhibits STING signaling by disrupting TBK1-STING interactions, blocking IFN-β secretion and enabling immune evasion. Preclinical studies in trastuzumab-resistant models demonstrate that combining STING agonists (e.g., ADU-S100) with HER2-targeted antibody-drug conjugates (e.g., DS-8201) significantly reduces the proportion of CD44+/CD24− cancer stem cells and enhances dendritic cell cross-presentation to promote CD8+ T-cell infiltration. The therapeutic challenges of TNBC stem from its high heterogeneity and immunosuppressive microenvironment. Current research focuses on manganese-based nanoagonists (e.g., BMP-Au), which activate the cGAS-STING pathway by inducing mitochondrial DNA release and synergize with radiotherapy to enhance immunogenic cell death, promoting CD8+ T-cell infiltration and significantly inhibiting TNBC lung metastasis. Combined with radiotherapy, this approach downregulates PD-L1 expression in the TME, reduces immunosuppressive myeloid-derived suppressor cells, and enhances antitumor immune responses. Additionally, GSH-responsive manganese oxide nanocubes activate both AMPK and STING pathways, inducing ferroptosis and immune responses to overcome chemoresistance. Mn²^+^release further activates the cGAS-STING pathway, promoting IFN-I secretion and APC function. However, studies also reveal that STING agonists may induce expansion of PD-L1^high^ monocytes via the IRF3-IFN-I axis, fostering an immunosuppressive microenvironment. TLR2 agonist pretreatment reprograms STING signaling by inducing K63 ubiquitination of STING, promoting its interaction with TRAF6, suppressing the IRF3-IFN-I axis, and activating the NF-κB pathway, thereby converting monocytes into PD-L1^low/–^ antitumor phenotypes. In breast cancer mouse models, combined TLR2/STING agonist therapy significantly inhibits tumor growth and induces systemic antitumor immunity. This mechanism has been validated in TNBC models (e.g., 4T1 breast cancer), where the combined strategy improves tumor suppression rates by over threefold and reduces lung metastasis. Nevertheless, hypoxia-induced metabolic reprogramming (e.g., ADSL-mediated fumarate accumulation) competitively inhibits cGAS activity, blocking STING signaling, while protective autophagy further weakens chemosensitivity by lysosomal degradation of STING protein. Future research should focus on exploring the specific mechanisms of the cGAS-STING pathway in different breast cancer subtypes, developing precise biomarkers and diagnostic tools, optimizing modulator development and application strategies, investigating multidisciplinary comprehensive treatment models, and identifying effective ways to overcome tumor heterogeneity and drug resistance. This will provide more precise and effective treatment options for breast cancer patients, improve their prognosis, and enhance survival rates and quality of life.

**Table 1:** Overview of the mechanisms andfunctions of cGAS-STING in breast cancer.

First author	year	Breast cancer subtypes	Model	Targets	cGAS/STING pathway status	Conclusion
Zhang et al. (47)	2024	luminal	Human breast cancer cell lines, Nude mouse models	cGAS-STING pathway, AKT1	Inactivated	Inactivated cGAS-STING signaling contributes to endocrine resistance through a positive feedback loop with AKT1.
Cai et al. (55)	2024	HER2+	HER2+ breast cancer cell lines Human HER2+ BC samples, Humanized mouse models	STING, IRF3	Inactivated	Silencing of cGAS-STING pathway is a key determinant of immune escape in Herceptin-resistant BC.
Wang et al. (69)	2024	TNBC	Oxygen-supplying nano-radiosensitizer	FePt alloy, MnO nanocrystals	Activated	Oxygen-supplying nano-radiosensitizer enhances TNBC radioimmunotherapy by activating cGAS-STING.
Xu et al. (67)	2024	TNBC	C5-AFt NPs	cGAS-STING	Activated	C5-AFt NPs inhibit TNBC growth and metastasis via ferroptosis and cGAS-STING activation.
Kim et al. (70)	2024	TNBC	Human TNBC cells	CBD, cGAS-STING	Activated	CBD enhances PD-L1 expression and Atezolizumab efficacy via cGAS-STING activation.
Zhong et al. (72)	2023	TNBC	Mouse breast cancer cell lines	GSDME, cGAS-STING	Activated	Nanodrug induces pyroptosis and activates cGAS-STING for enhanced TNBC therapy.
Zhou et al. (71)	2025	TNBC	Cu-ZnO2@PDA nanoplatforms	cGAS-STING	Activated	Zinc-copper nanoplatform enhances TNBC immunotherapy via cGAS-STING activation.
Liu et al. (66)	2022	TNBC	Human TNBC cell lines	cGAS-STING	Activated	cGAS-STING promotes TNBC cell survival under stress, offering new treatment opportunities.
Chen et al. (45)	2024	TNBC	Primary breast cancer samples (n=380), TCGA and METABRIC cohorts	STING, pTBK1, pSTAT1	Activated	cGAS-STING pathway is highly expressed in TNBC and is associated with genomic instability and immune cell infiltration.
Zhang et al. (51)	2025	BRCA	TAMs cell lines, Mouse models	cGAS, STING, TBK1, IRF3	Activated/Inactivated	Targeting cGAS-STING pathway for reprogramming TAMs shows promise in enhancing anti-tumor immunotherapy.
Ka et al. (37)	2023	BRCA	MMTV-PyMT mouse model, Breast cancer cell lines (MDA-MB-231, SKBR3)	NR1D1, cGAS, STING, IFN	Activated	NR1D1 enhances anti-tumor immunity via activating cGAS-STING pathway and promoting CD8+ T-cell responses.
Totis et al. (38)	2024	BRCA	An *in vitro* 4T1 breast cancer model	dsDNA、IFN-β	Activated	Carbon ions induce higher yields of cytoplasmic dsDNA fragments per unit dose compared to photons, and the release of interferon-β increases with increasing radiation dose.
Liu et al. (36)	2024	BRCA	83 breast cancer patients	STING, CD68	STING expression is lower in breast cancer tissue than in adjacent tissue	STING and CD68 are linked to breast cancer progression. STING activation may enhance TAMs function. STING agonists could improve immune therapy response.
Qin et al. (39)	2025	BRCA	Breast cancer cell lines and mouse models	PCK1, cGAS-STING	Inhibited by PCK1	PCK1 suppresses cGAS-STING activation, promoting immune evasion. Blocking PCK1 combined with anti-PD-1 therapy is effective. PCK1 S151 phosphorylation correlates negatively with patient survival.
